# Viral Vector-Based *Chlamydia trachomatis* Vaccines Encoding CTH522 Induce Distinct Immune Responses in C57BL/6J and HLA Transgenic Mice

**DOI:** 10.3390/vaccines12080944

**Published:** 2024-08-22

**Authors:** Giuseppe Andreacchio, Ylenia Longo, Sara Moreno Mascaraque, Kartikan Anandasothy, Sarah Tofan, Esma Özün, Lena Wilschrey, Johannes Ptok, Dung T. Huynh, Joen Luirink, Ingo Drexler

**Affiliations:** 1 Institute of Virology, Universitätsklinikum Düsseldorf, 40225 Düsseldorf, Germany; giuseppe.andreacchio@med.uni-duesseldorf.de (G.A.);; 2 R&D Department, Abera Bioscience AB, 75184 Uppsala, Sweden

**Keywords:** modified vaccinia virus Ankara, *Chlamydia trachomatis*, vaccine, cellular mediated immunity

## Abstract

*Chlamydia trachomatis* remains a major global health problem with increasing infection rates, requiring innovative vaccine solutions. Modified Vaccinia Virus Ankara (MVA) is a well-established, safe and highly immunogenic vaccine vector, making it a promising candidate for *C. trachomatis* vaccine development. In this study, we evaluated two novel MVA-based recombinant vaccines expressing spCTH522 and CTH522:B7 antigens. Our results show that while both vaccines induced CD4^+^ T-cell responses in C57BL/6J mice, they failed to generate antigen-specific systemic CD8^+^ T cells. Only the membrane-anchored CTH522 elicited strong IgG2b and IgG2c antibody responses. In an HLA transgenic mouse model, both recombinant MVAs induced Th1-directed CD4^+^ T cell and multifunctional CD8^+^ T cells, while only the CTH522:B7 vaccine generated antibody responses, underscoring the importance of antigen localization. Collectively, our data indicate that distinct antigen formulations can induce different immune responses depending on the mouse strain used. This research contributes to the development of effective vaccines by highlighting the importance of careful antigen design and the selection of appropriate animal models to study specific vaccine-induced immune responses. Future studies should investigate whether these immune responses provide protection in humans and should explore different routes of immunization, including mucosal and systemic immunization.

## 1. Introduction

Sexually transmitted diseases (STDs) continue to pose a global threat to sexual and reproductive health, impacting people’s daily life. According to the World Health Organization (WHO), over one million STDs are diagnosed worldwide each day, with *Chlamydia trachomatis* (*C. trachomatis*) accounting for approximately 130 out of 350 million cases in 2021 [1]. *C. trachomatis* is an obligate intracellular bacterium that can cause urogenital infection, lymphogranuloma venereum, and eye trachoma [2]. Such different outcomes depend on the specific serovars, from A to L, which are defined by the isoform of the chlamydial Major Outer Membrane Protein (MOMP). Women with *C. trachomatis* infection may face severe complications, such as pelvic inflammatory disease, ectopic pregnancy, endometritis, and infertility, while in men, it may cause urethritis and epididymitis [3]. Additionally, *C. trachomatis* is associated with a high risk of infection with other pathogens, such as Human Immunodeficiency Virus (HIV) [4,5]. Prophylaxis is the most advisable regimen against *C. trachomatis* since antibiotic efficiency is limited due to increasing resistance and does not prevent infection. Unfortunately, patients who have recovered from infection do not exhibit substantial evidence of long-lasting immunity, making them vulnerable to reinfection [6,7,8]. The situation is further complicated by the fact that the majority of infections are asymptomatic, with approximately 80% of women and 50% of men being affected. This factor could potentially have a significant impact on the transmission of *C. trachomatis* [9].

Despite the many attempts to develop prophylaxis against *C. trachomatis*, no commercial vaccine is currently available [10,11,12]. In a first-in-human clinical trial, a protein-based vaccine called CTH522, adjuvanted with CAF01^®^, demonstrated the ability to induce neutralizing IgG and IgA in serum and in the female reproductive tract of women [13]. CTH522 is a chimeric protein that combines a partial sequence of the MOMP from serovar D (aa 56–349) with the external VD4 domain from serovars E (aa 282–349), F (aa 283–351), and G (aa 283–351), which represent the most prevalent serovars [14,15,16]. In the B6C3F1/OlaHsd (H-2b, -2k) mouse model, immunization with CTH522 elicited CD4^+^ T cells and antibody response that offered protection against *C. trachomatis* infection [17,18,19]. However, the contribution of the CD8^+^ T-cell response was not extensively explored. The T-cell response is widely considered a critical player in protection against *C. trachomatis* infection [20,21]. In humans, increased recruitment of CD4^+^ and CD8^+^ T cells to the genital tract was observed in cervical secretions from Chlamydia-positive women [22]. In addition, resistance to Chlamydia infection and ascension of bacteria to the upper genital tract has been associated with a higher median frequency (approximately ^+^4.3%) of CD8^+^ T-cell responses in women with lower genital tract infections [23]. Clinical trials in non-human primates have highlighted the importance of T cells in vaccination strategies against *C. trachomatis* [24]. The depletion of CD8^+^ T cells in non-human primates followed by vaccination against the trachoma *C. trachomatis* serovar A resulted in a lack of protection in challenging experiments [25]. Previous research has shown that chlamydial infection in mice hampers the induction of memory CD8^+^ T-cell responses due to an excess of pro-inflammatory cytokines and alterations in the PD-1/PD-L1 immunomodulatory pathway [26,27]. Specifically, PD-L1 shows increased expression in uterine epithelial cells and dendritic cells in the draining lymph nodes following both primary and secondary chlamydial infection. This elevated expression results in a shift of Chlamydia-specific CD8^+^ T cells toward a central memory (Tcm) phenotype [28]. Tcm cells typically migrate through the secondary lymphoid organs and lymphatic vessels but lack efficient mechanisms for homing to or for effector functions in peripheral tissues [29]. This suggests an important role for local memory CD8^+^ T cells for protection against Chlamydia.

Recent work has suggested a role for CD8^+^ T cells in tissue damage of the genital tract during chlamydial infection in the OT-I mouse model [30]. In OT-I mice, the majority of CD8^+^ T-cell receptors are genetically modified to recognize only the H-2Kb-restricted ovalbumin peptide SIINFEKL [31]. As a result, they are unable to recognize chlamydial antigens, resulting in the absence of Chlamydia-specific CD8^+^ T cells. Notably, OT-I mice did not exhibit hydrosalpinx after intravaginal infection with Chlamydia [32]. In addition, adoptive transfer of spleen or mesenteric lymph-node-derived CD8^+^ T cells from Chlamydia-infected C57BL/6J mice to Chlamydia-infected OT-I mice appeared to exacerbate tissue damage in the OT-I mice. However, considering the compromised protective function of CD8^+^ T cells induced by chlamydial infection, as discussed above, this approach may potentially underestimate the actual role of CD8^+^ T cells in the protection against Chlamydia. In fact, it appears that bystander non-protective CD8^+^ and CD4^+^ T cells, recruited to the site of chlamydial infection through CXCR3, play a pivotal role in Chlamydia-induced immunopathology [33]. Yet, mice lacking CXCR3 and CCR5 have increased susceptibility to *C. trachomatis* infection [34]. In CXCR3 and CCR5 knock-out mice, Chlamydia-specific CD4^+^ T cells fail to migrate and be retained in genital tract tissues, resulting in reduced clearance of infection compared to wild-type mice. Collectively, the exact role of CD8^+^ T cells in the immunopathology of *C. trachomatis* infection remains elusive, and whether a vaccine is capable of inducing protective CD8^+^ T cells has not been clearly defined.

Modified Vaccinia virus Ankara (MVA), a poxvirus with greatly reduced virulence, which lost the ability to replicate in most mammalian cells, has been widely employed as a vaccine vector in both preclinical and clinical trials for various diseases, both infectious and not [35]. This vector is considered cost-effective, safe, and highly immunogenic, as recombinant MVA vaccines can generate robust humoral and cellular immune responses against various pathogens [36]. Therefore, MVA holds promise as a candidate vector for developing a vaccine against *C. trachomatis*.

In this study, we investigated the immunogenicity of novel *C. trachomatis* vaccine candidates based on the highly attenuated MVA. The viral vector has been genetically modified to express three distinct antigens, either CTH522, spCTH522, or CTH522:B7, designated MVA-CTH522, MVA-spCTH522, and MVA-CTH522:B7, respectively. Compared to CTH522, the spCTH522 construct includes the N-terminal sequence of MOMP (aa 1–55) with the aim to study the possible presence of T-cell epitopes within the MOMP. The CTH522:B7 construct is a cell membrane-anchored form of CTH522. To allow for the localization of CTH522 at the cell surface, the N-terminus of the antigen was fused to the signal sequence of the murine IgG κ-chain [37], while the C-terminus was extended with the transmembrane region and cytoplasmic domain of murine CD80, also called B7-1, thereby anchoring this protein to the cell surface membrane [38]. For both antigens, the respective modifications increased the stability of CTH522 significantly.

## 2. Materials and Methods

### 2.1. Mice

C57BL/6J mice were obtained from Janvier. A2.DR1 mice [39], transgenic for HLA-A*02:01/Db and for HLA-DRA*01:01 and HLA-DRB*01:01, were bred in-house at the “Zentrale Einrichtung für Tierforschung und wissenschaftliche Tierschutzaufgaben (ZETT, Central Institution for Animal Research and Scientific Animal Welfare, Düsseldorf, Germany)” under specific pathogen-free (SPF) conditions, following institutional guidelines, and they were routinely screened for the correct genotype. Mice were provided drinking water and a standardized mouse diet ad libitum. Female mice aged between 8 and 12 weeks were used in this study. After vaccination, the animals were kept in IVC cages (individually ventilated cages) and subjected to daily clinical assessment (appearance, posture, coat, behavior). At sacrifice, the mice were painlessly euthanized by cervical dislocation, and organs, blood, and vaginal washes were collected. Euthanasia did not require anesthesia.

### 2.2. Cells

HeLa (CCL-2) and UMNSAH/DF-1 (CRL-12203) cells were purchased from American Type Culture Collection (ATCC). All cells were cultured in RPMI 1640 medium (Invitrogen) containing inactivated 10% FBS (PAN Biotech) at 37 °C in 5% CO_2_.

### 2.3. Plasmid Construction and Recombinant Virus Generation

DNA sequences encoding CTH522, spCTH522, or CTH522:B7 antigens (Supplementary Table S1) were codon optimized for eukaryotic expression and enhanced ribosomal binding and protein production. The DNA sequences were synthetized by GeneArt^®^ (Thermo Fisher Scientific). To create MVA transfer plasmids carrying either the CTH522, spCTH522 or CTH522:B7 expression cassette, the corresponding DNA sequences were PCR amplified using primers designed to generate BamHI-CTH522-AflII, BamHI-spCTH522-AflII, and BamHI-CTH522:B7-AflII fragments, respectively. The purified PCR products were then cloned into the MVA transfer plasmid pVI-PmH5-dVI using BamHI and AflII restriction sites. The expression of recombinant antigens was controlled by the modified PH5-vaccinia virus strong early and late promoter [40]. The generation of recombinant MVA was achieved through a BAC recombinant system, as previously described [41]. Briefly, the transgene expression cassette, after being linearized by PacI restriction enzyme, was inserted into the deletion VI region of the MVA genome. This was achieved through a two-step red-recombination protocol in GS1783 E. coli containing the GFP-expressing MVA-BAC genome, resulting in the generation of recombinant MVA-BAC. The recombinant BAC was subsequently extracted and purified. In order to generate recombinant viruses, the recombinant BAC was transfected in DF-1 cells, followed by infection with Rabbit Fibroma Virus (RFV), which provided the initial transcription machinery to enable the replication of recombinant MVA. Finally, DF-1 cells were repeatedly infected with the recombinant MVAs. This process allowed for the removal of the BAC backbone by self-excision properties of our BAC system. In addition, the passaging steps in DF-1 cells ensured the clearance of the helper virus. Finally, infection of DF-1 cells in limiting dilution steps of recombinant MVAs was carried out to obtain single clones of recombinant MVAs.

### 2.4. Viruses

The non-recombinant MVA (here referred to as MVA-WT), which served as non-recombinant control, and the recombinant MVAs expressing CTH522, spCTH522, and CTH522:B7, were generated using the BAC-based recombination technique described above. All MVAs were propagated and titrated on DF-1 cells, as described elsewhere [42].

### 2.5. PCR Analysis of Recombinant MVAs

The generation and purity of MVA-BACs and recombinant MVA-CTH522, MVA-spCTH522, and MVA-CTH522:B7 were assessed through PCR analysis. Oligonucleotides DelVI-L (5′-CTCCGCATCTAGTTGATATTCCAACCTCTT-3′) and DelVI-R (5′-CCTGGACATTTA-GTTTGAGTGTTCCTGAAT-3′), designed to anneal to the MVA left and right flanking regions of Deletion VI, respectively, were used for PCR amplification of the respective CTH522-, spCTH522-, or CTH522-B7-containing expression cassettes. Additionally, DNA sequencing was performed to confirm the identity of the CTH522, spCTH522, and CTH522:B7 sequences.

### 2.6. SDS-PAGE and Western Blot

To test the synthesis of the CTH522, spCTH522, and CTH522:B7 antigens, monolayers of HeLa cells were mock-infected or MVA-infected at MOI of 5 with MVA-WT, MVA-CTH522, MVA-spCTH522, or MVA-CTH522:B7. At specific time points, post-infection cells were treated with RIPA lysis buffer, and 25–30 μg of total extracted proteins was loaded on and separated in 10% SDS-PAGE gels and transferred to nitrocellulose membranes. Membranes were blocked with 5% low-fat milk powder dissolved in TRIS-buffered saline supplemented with 0.1% Tween-20 (TBST) for 1 h at room temperature in a horizontal shaker. Membranes were incubated with a mouse anti-*C. trachomatis* L2 species-specific MOMP antibody (clone MAb6ciii, Chlamydia Bio-bank; diluted 1:10,000) to detect spCTH522 and CTH522:B7 proteins. As controls, mouse anti-β-actin antibody (Sigma; diluted 1:10,000) and rabbit anti-VACV H3 antibody (diluted 1:500) were used. Primary antibodies were incubated with membranes for 1 h at room temperature or overnight at 4 °C in horizontal shaker. Horseradish peroxidase-conjugated goat anti-rabbit or anti-mouse IgG (Jackson, diluted 1:5000 and 1:3000, respectively) were used as secondary antibodies and incubated with membranes for 1 h at room temperature. Chemiluminescence detection was carried out with Super Signal West Dura Chemiluminescent Substrate (ThermoFisher Scientific, Waltham, MA, USA).

### 2.7. Immunofluorescence Microscopy and Flow Cytometry

Immunofluorescence experiments were performed on HeLa cells at 6 h post-infection. Non-permeabilized infected HeLa cells were fixed with 4% formaldehyde for 15 min at room temperature. Cells were then washed with PBS and stained with WGA probe conjugated to the red fluorescent dye Alexa Fluor 594 (Invitrogen, Waltham, MA, USA; diluted 1:200) and mouse monoclonal antibody against MOMP protein (Chlamydia Biobank; diluted 1:2000) to label the cell membrane and CTH522 proteins, respectively. Cells were incubated with probe and primary antibody for 1 h at room temperature. Bound anti-MOMP was then identified using anti-mouse secondary antibodies conjugated to the Alexa Fluor 488 (green) fluorochrome (Invitrogen; diluted 1:200). Cell sections were imaged using a ZEISS LSM 880 confocal laser scanning microscope. For flowcytometry experiments, non-permeabilized infected HeLa cells were washed with PBS and stained with viability dye (eBioscience^TM^ Fixable Viability Dye eFluorTM 660) for 20 min on ice. Subsequently, cells were washed twice with staining buffer (1% BSA, 0.02% NaN_3_ in PBS) and stained for 1 h on ice with a mouse monoclonal antibody against MOMP protein (Chlamydia Biobank; diluted 1:2000). Bound anti-MOMP was then identified using anti-mouse secondary antibodies conjugated to the phycoerythrin (PE) (1:200; Jackson ImmunoResearch Europe Ltd., West Grove, PA, USA) for 30 min on ice. Cells were subsequently washed and fixed with 1% paraformaldehyde. Flow cytometry was performed using BD FACS Canto II (BD Biosciences, Heidelberg, Germany).

### 2.8. Immunization and Tissue Collection

C57BL/6J or A2.DR1 female mice were vaccinated intraperitoneally (i.p.) with 1 × 10^8^ IU (infectious units) of MVA-WT, MVA-spCTH522, or MVA-CTH522:B7 in 200 μL final volume with PBS. Vaccination was performed either as single vaccination or as prime/boost regimen with a boost i.p. at day 28. In the prime/boost regimen, mice received 1 × 10^7^ IU as priming dose and 1 × 10^8^ IU as boosting dose. Spleens were harvested 8 days after prime or 28 days after boost vaccination to measure T-cell responses using intracellular cytokine staining (ICS). Blood and vaginal washes with 100 uL of PBS supplemented with a cocktail of protease inhibitors (Roche) were collected 28 days after the boost and analyzed for antibody responses using ELISA.

### 2.9. Peptides

An spCTH522-specific overlapping peptide pool was designed and subsequently used in the immunogenicity analysis. Purified peptides of the pool (1 mg per vial) were obtained from Peptides&Elephants. Peptides spanned the entire spCTH522 protein as consecutive 15-mers peptides overlapped by 11 amino acids. Further peptides were used: B8_20−27_ (H2-Kb) TSYKFESV, OVA_257–264_ (H2-Kb) SIINFEKL, B5_46–60_ (I-Ab) FTCDQGYHSSDPNAV, OVA_323–339_ (I-Ab) ISQAVHAAHAEINEAGR, B8_20−27_ (HLA-A*02:01) TSYKFESV, M1_58–66_ (HLA-A*02:01) GILGFVFTL, A10_293–307_ (HLA-DR1) SMRYQSLIPRLVEFF, M1_17–30_ (HLA-DR1) SGPLKAEIAQRLED, MOMP^D^_282–290_ (HLA-A*02:01) NMFTPYIGV, MOMP^D^_200–209_ (HLA-A*02:01) ALWECGCATL.

### 2.10. Cellular-Specific Immune Response Analysis

Spleens were mashed on 70 µm cell strainer. To remove erythrocytes, the cells were incubated with lysis buffer (BD Pharm LyseTM^®^) for 1 min at room temperature. Subsequently, the cells were passed through another 70 μm cell strainer and counted using a Neubauer cell counting chamber. Then, 2 × 10^6^ cells were plated in 200 μL per well of a 96-well plate and incubated with spCTH522 pool peptides (either total pool or fractions of the pool), specific peptides, or control peptides at a concentration of 5 μg/mL, in the presence of 1 μg/mL of Brefeldin A (Merck), for 4–5 h.

### 2.11. Intracellular Staining

Cells were washed with PBS, and dead cells were stained with viability dye (eBioscienceTM Fixable Viability Dye eFluorTM 660) for 20 min on ice. Subsequently, cells were washed twice with staining buffer (1% BSA, 0.02% NaN_3_ in PBS) and stained for 30 min on ice with a combination of antibodies for surface markers (see below). Cells were then washed twice, fixed, and permeabilized for 15 min on ice using BD Cytofix/CytopermTM solution. After an additional washing step, cells were stained for 30 min in the dark on ice with a combination of antibodies for intracellular markers. Finally, cells were washed and fixed with 1% paraformaldehyde. Flow cytometry was performed using BD FACS Canto II (BD Biosciences, Heidelberg, Germany).

Antibodies: eFluor405 anti-mCD8 (Invitrogen), PE-Cy7 anti-mCD4 (Biolegend), PE anti-mCD107 (Biolegend), FITC anti-mIFNγ g (BD), APC-Cy7 anti-mTNFα (Biolegend), PerCP-Cy5.5 anti-mIL2 (BD).

### 2.12. Enzyme-Linked Immunosorbent Assay

MaxiSorp round-bottom 96-well plates (Nunc, Roskilde, Denmark) were coated with 100 µL of recombinant CTH522 (5 ug/mL) in 0.1 M carbonate/bicarbonate buffer (pH 9.6) overnight at 4 °C. The next day, the plates were washed three times with PBS containing 0.05% Tween-20 (PBST) and then blocked with 3% BSA/PBST for 1 h. Sera or vaginal washes from mice immunized with MVA-WT, MVA-spCTH522, and MVA-CTH522:B7 were serially diluted in 1% BSA/PBST, added to the wells, and incubated for 1 h. After three washes with PBST, the plates were incubated with alkaline phosphatase-conjugated goat anti-mouse total IgG, IgG1, IgG2b, IgG2c, IgG3, or IgA secondary antibodies (1:1000; Jackson ImmunoResearch Europe Ltd.) for 1 h. After three washes with PBST, p-nitrophenyl phosphate substrate (pNPP) (Sigma Aldrich, St. Louis, MO, USA) was used for color development for 30 min. The reaction was stopped by adding 2 M NaOH, and the absorbance was measured at 405 nm using a spectrophotometer microplate reader (Thermo Fisher Scientific). All procedures were performed at room temperature unless otherwise stated.

### 2.13. Statistical Analysis

Data handling, graphical visualizations, and analysis with either Kruskal–Wallis test followed by Dunn’s multiple comparisons unpaired *t* test with Welch’s correction or Mann–Whitney test were conducted using GraphPad Prism version 9.0.0. A *p*-value below 0.05 was considered significantly different. * *p* < 0.05, ** *p* < 0.01, *** *p* < 0.001, **** *p* < 0.0001. ns, non-significant.

## 3. Results

### 3.1. Generation and In Vitro Characterization of MVA-CTH522

Initially, we developed a vaccine candidate designated MVA-CTH522. This viral vector vaccine was constructed using the highly attenuated vaccinia virus strain MVA and expresses a codon-optimized CTH522 antigen. To generate recombinant MVA-CTH522, we inserted the recombinant CTH522 expression cassette into the deletion VI locus of the MVA-BAC genome (Supplementary Figure S1A). Purified single clones of MVA-CTH522 were verified to be free of helper virus, which was used in the co-infection/transfection process with recombinant BAC to produce recombinant MVA. In addition, MVA-CTH522 was tested for the absence of the BAC backbone (Supplementary Figure S1B). The expression of the CTH522 gene is regulated by the synthetic early-late promoter, PmH5. The correct insertion of the 1986 bp CTH522 expression cassette into the MVA-CTH522 genome was confirmed by PCR analysis (Supplementary Figure S1C).

To demonstrate the ability of MVA-CTH522 to express the respective recombinant antigen, we performed Western blot analysis using cell extracts obtained from human HeLa cells at various time points after infection. HeLa cells were either mock-infected or infected with MVA-CTH522 or MVA-WT. Specific antibodies against the MOMP of C. trachomatis serovar D were used. Surprisingly, no protein band was detected in the Western blot analysis (Supplementary Figure S1D). However, Western blot analysis showed correct synthesis of MVA-derived H3 protein, suggesting that the absence of detectable antigen was not due to the failure of the in vitro infection.

Given that CTH522 is an engineered construct designed for bacterial production, the antigen might be perceived as a foreign or misfolded protein upon MVA-mediated expression in mammalian cells and consequently be rapidly degraded. To test this hypothesis, proteasome inhibition followed by Western blot analysis was performed. HeLa cells were infected with an MOI of 5 of MVA-WT or MVA-CTH522 for 24 h. At 3 h post-infection (h.p.i.), cells were treated with 2 µM of the irreversible proteasome inhibitor epoxomicin. Notably, inhibition of the proteasome–ubiquitin system facilitated the detection of CTH522 protein at the in silico predicted size of approximately 53.5 kDa (Supplementary Figure S1E). These data show that CTH522 is subject to rapid degradation upon expression in mammalian cells after MVA infection.

### 3.2. Generation and In Vitro Characterization of MVA-spCTH522 and MVA-CTH522:B7

**Figure 1:**
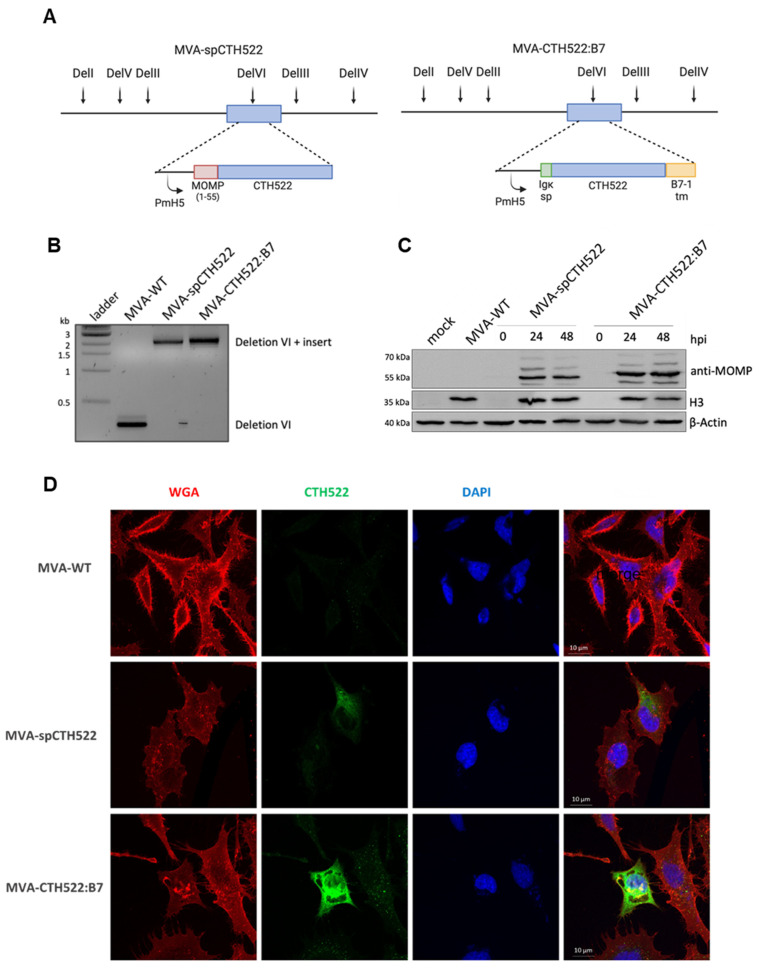
Construction, genetic characterization, recombinant antigen expression and localization of MVA-spCTH522 and MVA-CTH522:B7 (**A**) Schematic representation of MVA-spCTH522 (**left**) and MVA-CTH522:B7 (**right**) genomes. The location of the respective expression cassettes in the major deletion site VI (del VI) of the MVA genome is shown at the top. PmH5, modified early late H5 vaccinia virus promoter (**B**) Viral genomic DNA was extracted from stocks of non-recombinant MVA (MVA-WT), MVA-spCTH522 and MVA-CTH522:B7 and analyzed by PCR using delVI-specific primers. The PCR fragments obtained for the respective expression cassettes are expected to be 2151 bp in size for spCTH522 and 2256 bp for CTH522:B7 (**C**) HeLa cells were mock-infected or infected with MVA-WT, MVA-spCTH522 or MVA-CTH522:B7 (MOI = 5). Cells were lysed at 0, 24 or 48 hpi (MVA-spCTH522 and MVA-CTH522:B7) or at 48 hpi (mock and MVA-WT). Whole cell lysates were analyzed by Western blotting with antibodies against MOMP-SvD, displaying recombinant proteins with an expected size of ~57.4 kDa for spCTH522 and ~63.9 kDa for CTH522:B7. Vaccinia virus H3 protein and b-actin were used as infection and loading controls, respectively (**D**) CTH522:B7 is located at the cell membrane. Confocal laser microscopy immunofluorescence analysis (CLSM) to determine the location of spCTH522 and CTH522:B7 proteins in the infected cells. HeLa cells infected with MVA-spCTH522, MVA-CTH522:B7, or MVA-WT at MOI of 5 were fixed 6 h after infection, and non-permeabilized cells were stained with WGA probe conjugated to Alexa Fluor 594 (red) and a mouse monoclonal anti-MOMP antibody. Bound anti-MOMP was visualized using a mouse secondary antibody conjugated to Alexa Fluor 488 (green). Nuclei were stained with DAPI (blue). Scale bars: 10 µm.

Given the unstable nature of the CTH522 antigen when expressed by MVA, which could potentially affect its immunogenicity [43], we generated recombinant MVAs expressing modified versions of the CTH522 antigen, specifically spCTH522 or CTH522:B7. To create MVA-spCTH522 and MVA-CTH522:B7, we inserted the respective expression cassettes into the deletion VI locus of the MVA-BAC genome (Figure 1A). We confirmed the correct insertion of the 2151 bp long spCTH522 and 2256 bp long CTH522:B7 expression cassette into the genome of MVA-spCTH522 and MVA-CTH522:B7, respectively, using PCR analysis (Figure 1B).

Both MVA-spCTH522 and MVA-CTH522:B7 were able to express the respective recombinant antigens at the in silico predicted sizes, approximately 57.4 kDa for spCTH522 and 63.9 kDa for CTH522:B7, as demonstrated by Western blot analysis using cell extracts taken from human HeLa cells at different time points post-infection with either mock or MVA-spCTH522, MVA-CTH522:B7, or MVA-WT (Figure 1C). Interestingly, both modifications increased the stability of the CTH522 antigen.

To further assess whether spCTH522 and CTH522:B7 proteins could be detected on the cell surface, HeLa cells were infected with recombinant MVAs, and non-permeabilized cells were analyzed by confocal immunofluorescence microscopy using an antibody against MOMP protein and WGA probe to label the fixed cell surface (Figure 1D). Although the infected cell surface was clearly visible with the WGA probe, spCTH522 protein was weakly detected on the surface of MVA-spCTH522-infected cells. In contrast, we observed substantial localization of CTH522:B7 protein on the cell surface of MVA-CTH522:B7-infected cells. The surface localization of CTH522:B7 was confirmed by FACS analysis of HeLa cells infected with MVA-WT or MVA-CTH522:B7 using an antibody against MOMP protein (Supplementary Figure S2A,B). These results demonstrate that both spCTH522 and CTH522:B7 proteins were produced upon MVA infection and that CTH522:B7 was targeted to the cell surface.

### 3.3. MVA-spCTH522 and MVA-CTH522:B7 Induced Systemic CD4^+^ But Not CD8^+^ T-Cell Responses against CTH522 in C57BL/6J Mice

**Figure 2:**
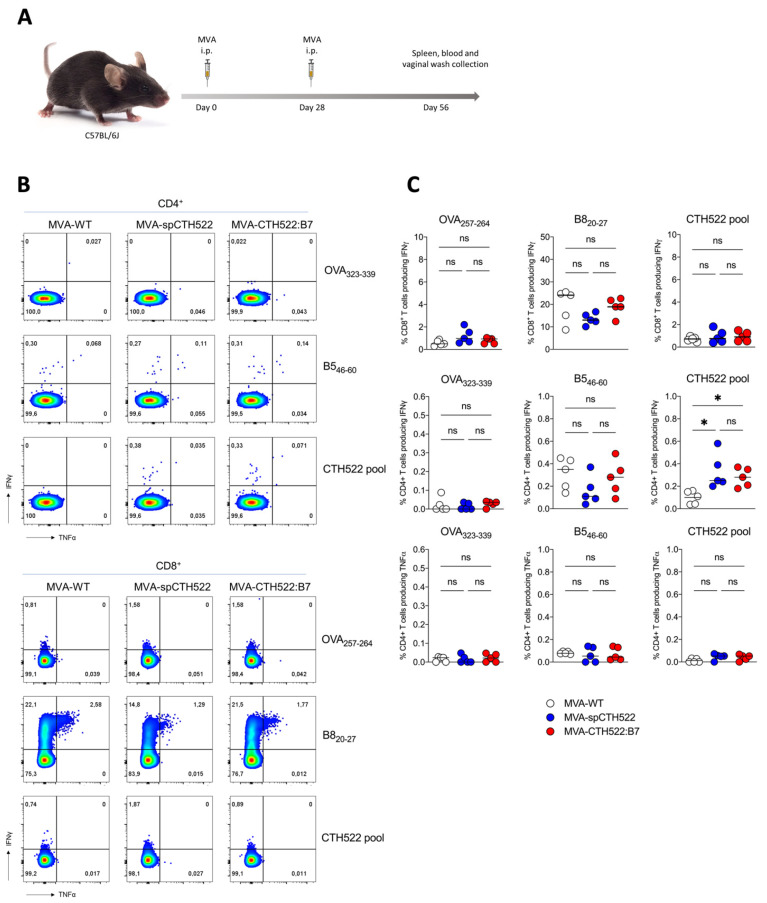
MVA-spCTH522 and MVA-CTH522:B7 induced CTH522-specific CD4^+^ but not CD8^+^ T-cell responses in C57BL/6J mice (**A**) Schematic representation of the experimental setting. C57BL/6J mice were immunized intraperitoneally with MVA-WT, MVA-spCTH522 or MVA-CTH522:B7 (n = 5 per group) on days 0 and boosted on day 28. Spleens were harvested on day 56 and splenocytes were restimulated with a pool of overlapping peptides spanning the full spCTH522 sequence. OVA-specific (OVA_257–264_, OVA_323–339_) and MVA-specific (B8_20−27_, B5_46–60_) peptides were used as negative and positive controls, respectively (**B**) Representative flow cytometry gates of cytokine-producing CD4^+^ (upper gates) and CD8^+^ T cells (lower gates) from the spleens of MVA-WT, MVA-spCTH522 or MVA-CTH522:B7 immunized mice (**C**) Frequencies of CD8^+^ T cells and CD4^+^ T cells producing IFNγ or TNFα were measured by flow cytometry. Each data point represents one mouse, and short horizontal lines geometrical mean. Kruskal–Wallis test followed by Dunn’s multiple comparisons was used to compare groups (ns, non-significant, * *p* < 0.05).

To explore the potential of MVA-spCTH522 and MVA-CTH522:B7 to elicit antigen-specific T-cell responses against C. trachomatis, we tested our two recombinant MVAs in C57BL/6J mice using a homologous prime/boost immunization regimen (Figure 2A). As T cells are known to have a protective role against C. trachomatis infection, we determined CTH522-specific CD4^+^ and CD8^+^ T-cell responses at 28 days post-boost, performing an ICS assay after stimulating splenocytes with an overlapping peptide library spanning the entire spCTH522 protein. Our results revealed that neither MVA-spCTH522 nor MVA-CHT522:B7 was able to elicit CTH522-specific CD8^+^ T-cell responses in the spleen. In contrast, mice immunized with either MVA, including MVA-WT, showed CD8^+^ T-cell responses against the MVA backbone-derived B8 peptide contained in recombinant and non-recombinant MVA (Figure 2B,C), which is a well-established H2-Kb-restricted epitope conserved among various vaccinia virus strains [44]. This indicates that the lack of response was not due to an immunization failure. In contrast, both MVA-spCTH522 and MVA-CTH522:B7 induced CTH522-specific CD4^+^ T cells in the spleen, while no detectable CTH522-specific CD4^+^ T-cell response was observed in MVA-WT-immunized mice. This indicates the absence of cross-reactivity between the MVA-specific CD4^+^ T-cell response and CTH522 antigen. There was no CD4^+^ and CD8^+^ T-cell response against MHC class II and I-restricted OVA peptides, respectively, which served as negative controls.

### 3.4. MVA-CTH522:B7 But Not MVA-spCTH522 Induced Humoral Responses in C57BL/6J Mice

**Figure 3:**
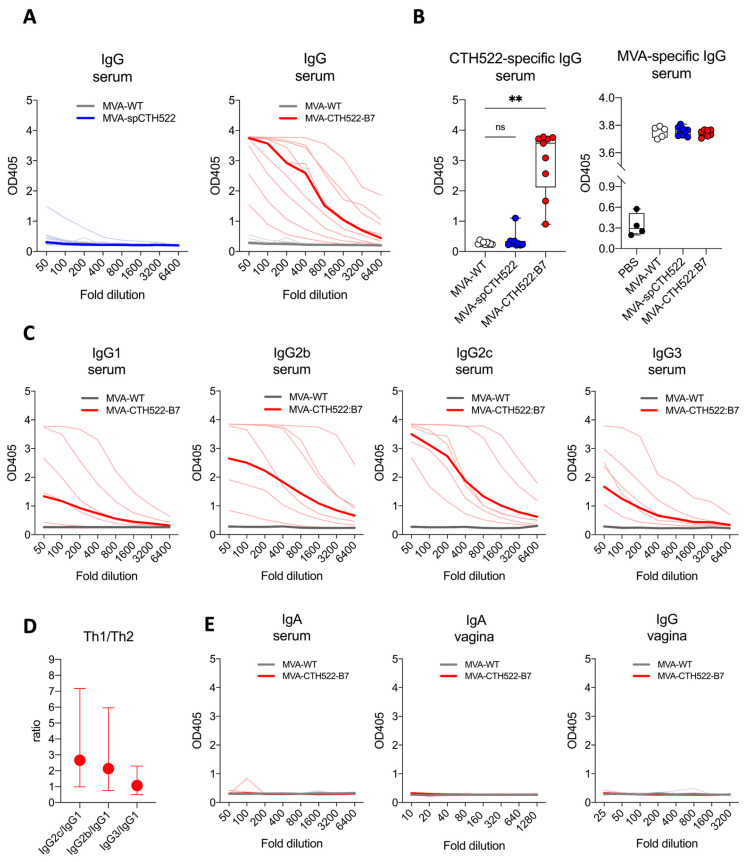
MVA-CTH522:B7 but not MVA-spCTH522 induced humoral responses in C57BL/6J mice. C57BL/6J mice (n = 9 per group) were immunized as shown in Figure 2A (**A**) Serum samples from MVA-spCTH522 (**left**) and MVA-CTH522:B7 (**right**) immunized mice were serially diluted and added to CTH522-coated plates. Antigen-specific IgG was analyzed by enzyme-linked immunosorbent assay (ELISA). Single thin lines represent each individual mouse, thick lines represent the geometrical mean of the optical density values at each dilution step (**B**) Individual OD405 values for IgG obtained at a serum dilution of 1:100 for CTH522-specific antibodies (**left**) and at a dilution of 1:500 for MVA-specific antibodies (**right**). For statistical analysis, Kruskal–Wallis test followed by Dunn’s multiple comparisons was used to compare CTH522-specific antibodies between groups (ns, non-significant, ** *p* < 0.005) (**C**) Levels of CTH522-specific IgG subtypes (IgG1, IgG2b, IgG2c and IgG3) in serum were determined by ELISA from mice immunized with either MVA-CTH522:B7 or MVA-WT. Single thin lines represent each individual mouse, thick lines represent the geometrical mean of the optical density values at each dilution step (**D**) Ratio of OD405 values were calculated at 1:100 dilution for IgG2b, IgG2c or IgG3 to IgG1. Values are shown as geometric mean with geometric SD (**E**) Levels of CTH522-specific IgG and IgA antibodies were determined by ELISA in serum or vaginal washes from mice immunized with MVA-CTH522:B7 or MVA-WT.

To evaluate the humoral response induced by MVA-spCTH522 and MVA-CTH522:B7, CTH522-specific antibodies were measured in C57BL/6J mice immunized with MVA-WT, MVA-spCTH522, or MVA-CTH522:B7. Mice were vaccinated in a prime/boost regimen, and blood and vaginal washings were collected four weeks post-boost, as mentioned above (Figure 2A). Mice exhibited humoral responses to CTH522 after immunization with MVA-CTH522:B7, while no response was observed with MVA-spCTH522 (Figure 3A,B). There was no detectable CTH522-specific response for MVA-WT, confirming the specificity of the ELISA and absence of cross-reactivity between the MVA-specific antibody response and CTH522. In contrast, mice immunized with either MVA-WT, MVA-spCTH522, or MVA-CTH522:B7 showed antibody response against MVA backbone antigens (Figure 3B). This indicates that the absence of antibodies against CTH522 in MVA-spCTH522-vaccinated mice was not due to immunization failure.

Further examination of the CTH522-specific antibody response in MVA-CTH522:B7-immunized mice revealed a diverse profile of IgG subclasses, with IgG2b and IgG2c being significantly enhanced compared to IgG1 and IgG3, indicating that MVA-CTH522:B7 induced a Th-1-biased humoral response (Figure 3C,D). On the other hand, no CTH522-specific IgA antibodies were detected in the serum of the immunized mice (Figure 3E). To assess the antibody response in the genital tract, antibodies were measured in vaginal washings. However, no IgG or IgA was detected in the vaginal washings (Figure 3E).

### 3.5. MVA-CTH522:B7 Induced T-Cell and Antibody Responses in HLA Transgenic Mice

**Figure 4:**
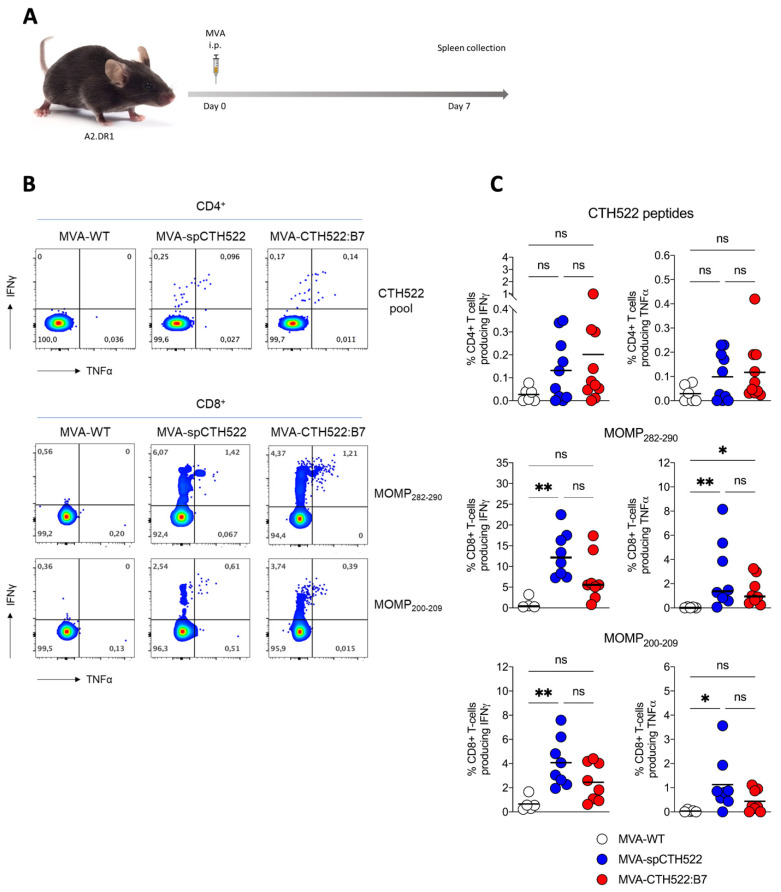
A single dose of MVA-spCTH522 and MVA-CTH522:B7 induces MOMP-specific CD8^+^ T cell immune responses in HLA transgenic mice (**A**) Schematic representation of the experimental setting. A2.DR1 mice were immunized intraperitoneally with MVA-WT (n = 6), MVA-spCTH522 (n = 7), or MVA-CTH522:B7 (n = 7) on days 0. Spleens were harvested on day 7 and splenocytes were restimulated with a pool of overlapping peptides spanning the full spCTH522 sequence to determine CD4^+^ T-cell responses or pulsed with MOMP_282–290_ or MOMP_200–209_ peptides to determine CD8^+^ T-cell responses by ICS assay (**B**) Representative flow cytometry gates of cytokine-producing CD4^+^ (upper gates) and CD8^+^ T cells (lower gates) from the spleens of MVA-WT, MVA-spCTH522 or MVA-CTH522:B7 immunized mice (**C**) Total percentage of CTH522-specific CD4^+^ T cells (upper graphs), MOMP_282–290_- (middle graphs) and MOMP_200–209_- (lower graphs) specific CD8^+^ T cells producing IFNγ or TNFα. Each data point represents one mouse, and short horizontal lines geometrical mean. Kruskal–Wallis test followed by Dunn’s multiple comparisons was used to compare groups (ns, non-significant, * *p* < 0.05, ** *p* < 0.005).

Given that we aim to develop a human vaccine with particular focus on the CD8^+^ T-cell response, we decided to employ a transgenic mouse model that allows us to investigate the vaccine-induced T-cell responses in HLA-restricted manners, here referred to as A2.DR1 mice. These mice are knock-out for the endogenous major histocompatibility complexes (MHCs) I and II and transgenic for HLA-A*02:01/Db and HLA-DR1 as the only sources of MHC-I and MHC-II, respectively [39]. When we immunized A2.DR1 mice in a single vaccination regimen (Figure 4A), we observed that both MVA-spCTH522 and MVA-CTH522:B7 did not induce strong CD4^+^ T-cell responses specific to the spCTH522 overlapping peptide pool (Figure 4B,C). In contrast, by using the overlapping peptides we identified two HLA-A*02:01-restricted epitopes within the CTH522 protein sequence that were able to induce CD8^+^ T-cell responses (Figure 4B,C). The first epitope was the 9-mer NMFTPYIGV, which corresponds to MOMP_282–290_. This epitope was previously described as a dominant CD8^+^ T-cell-specific epitope in PBMCs of a cohort of C. trachomatis-positive patients [45]. In addition, we identified a second novel epitope, the 10-mer ALWECGCATL, which corresponds to MOMP_200–209_.

**Figure 5:**
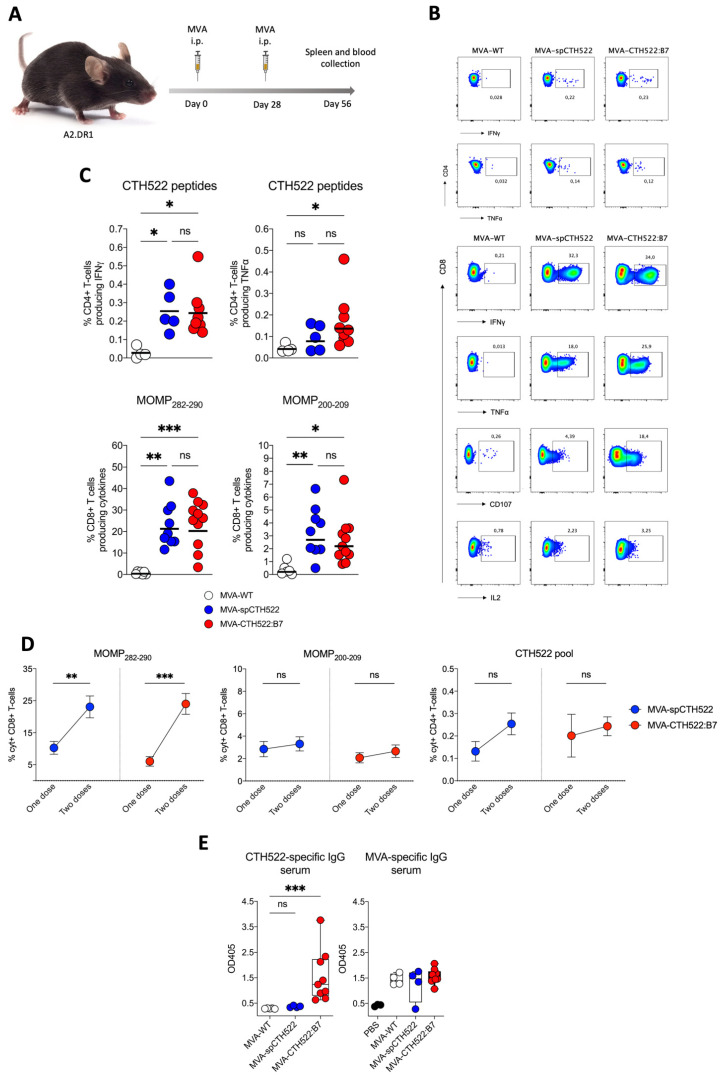
A boosting dose of MVA-spCTH522 or MVA-CTH522:B7 enhanced the magnitude of T-cell responses in HLA transgenic mice (**A**) Schematic representation of the experimental setting. A2.DR1 mice were immunized intraperitoneally with MVA-WT (n = 5), MVA-spCTH522 (n = 9) or MVA-CTH522:B7 (n = 11) on days 0 and boosted at day 28. Spleens were harvested on day 56 and splenocytes were restimulated with either a pool of overlapping peptides spanning the full spCTH522 sequence or HLA-A*0201-restricted MOMP_282–290_ or MOMP_200–209_ peptides. Activation of CD4^+^ and CD8^+^ T cells was measured by ICS assay (**B**) Representative flow cytometry gates of cytokine-producing CD4^+^ (upper gates) and CD8^+^ T cells (lower gates) from the spleens of MVA-WT, MVA-spCTH522 or MVA-CTH522:B7 immunized mice (**C**) Overall percentage of CTH522-specific CD4^+^ T cells producing IFNγ or TNFα (upper graphs) after stimulation with the peptide pool and percentage of MOMP_282–290_- and MOMP_200–209_- specific CD8^+^ T cells (lower graphs) producing IFNγ, TNFα, CD107 and IL2 (cytokines). For statistical analysis, Kruskal–Wallis test followed by Dunn’s multiple comparisons was used to compare groups (**D**) Comparison of the CD8^+^ and CD4^+^ T cells at 7 days after prime (see Figure 4C) or 28 days after boost (see Figure 5C) with MVA-spCTH522 or MVA-CTH522:B7. Statistical analysis was performed using unpaired *t* test with Welch’s correction (**E**) A2.DR1 mice were immunized as shown in Figure 5A with MVA-WT, MVA-spCTH522, or MVA-CTH522:B7, and antigen-specific total IgG was analyzed by enzyme-linked immunosorbent assay (ELISA). Individual OD405 values for IgG were obtained at a serum dilution of 1:100 for CTH522-specific antibodies (**left**) and MVA-specific antibodies (**right**). For statistical analysis, Kruskal–Wallis test followed by Dunn’s multiple comparisons was used to compare CTH522-specific antibodies between groups (ns, non-significant, * *p* < 0.05, ** *p* < 0.005, *** *p* < 0.0005).

In prime/boost regimens, the MVA booster is known to increase T-cell memory responses [46]. To assess whether a second dose of MVA-spCTH522 and MVA-CTH522:B7 would increase the T-cell response in A2.DR1 mice, we immunized mice in a homologous prime/boost vaccination regimen and analyzed the T-cell response 28 days after the boost (Figure 5A). Both MVA-spCTH522 and MVA-CTH522:B7 induced a comparable CD4^+^ and CD8^+^ T-cell response specific to CTH522 and MVA (Figure 5B,C and Supplementary Figure S3). A significant increase in the magnitude of CD8^+^ T cells specific to MOMP_282–290_ was observed 28 days after the boost dose compared to the magnitude of CD8^+^ T cells 7 days after the single-dose vaccination. However, no statistical difference in the magnitude of CD8^+^ T cells specific to MOMP_200–209_ and in the magnitude of CD4^+^ T cells specific to CTH522 was observed between the two vaccination regimens (Figure 5D).

Our previous results showed that only MVA-CTH522:B7 was able to induce CTH522-specific antibody responses in C57BL/6J mice. To evaluate whether MVA-CTH522:B7 was able to induce CTH522-specific antibodies in A2.DR1 mice, mice were immunized with MVA-WT, MVA-spCTH522, or MVA-CTH522:B7 in a prime/boost regimen, as mentioned above (Figure 5A). Mice exhibited humoral responses to CTH522 after immunization with MVA-CTH522:B7, while there were no detectable CTH522-specific IgG in MVA-WT and MVA-spCTH522-immunized mice. In contrast, mice immunized with either MVA-WT, MVA-spCTH522, or MVA-CTH522:B7 generated antibodies against MVA backbone antigens, confirming the specificity of the ELISA and absence of cross-reactivity between the MVA-specific antibody response and CTH522 in A2.DR1 mice (Figure 5E).

### 3.6. MVA-spCTH522 and MVA-CTH522:B7 Induced Multifunctional CD8^+^ T Cell in HLA Transgenic Mice

**Figure 6:**
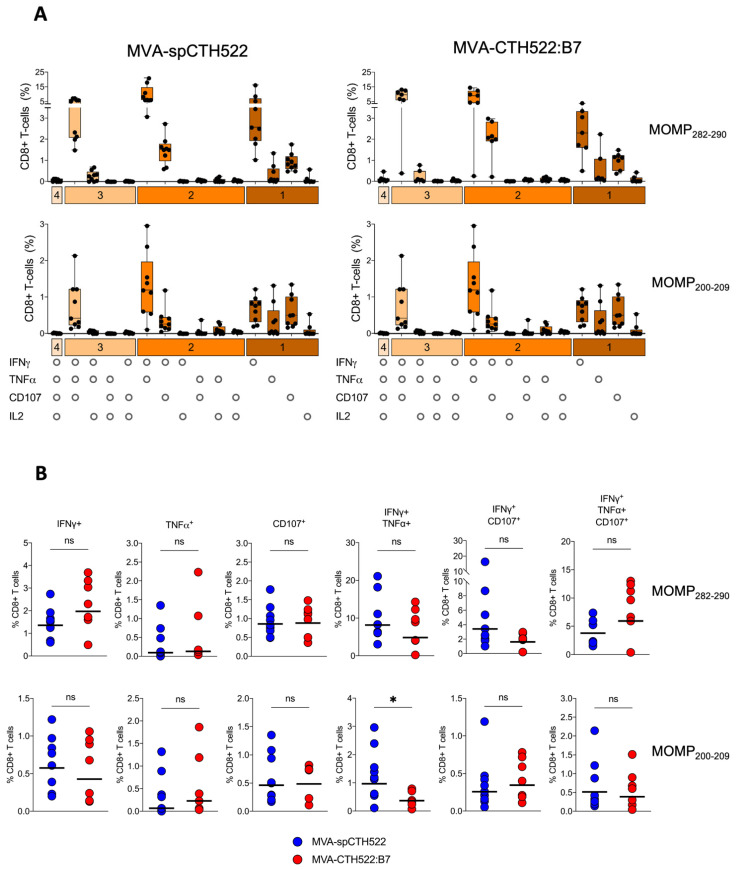
MVA-spCTH522 and MVA-CTH522:B7 elicit multifunctional CD8^+^ T-cell responses. Splenocytes from A2.DR1 mice immunized intraperitoneally with MVA-spCTH522 or MVA-CTH522:B7 (Figure 5A) were incubated with either the HLA-A*02:01-restricted MOMP_282–290_ or MOMP_200–209_ peptides (**A**) Polyfunctional profiles of MOMP_282–290_- and MOMP_200–209_-specific CD8^+^ T cells in MVA-spCTH522 (**left** graphs) and MVA-CTH522:B7 (**right** graphs) immunized mice. All possible combinations of cytokines tested are plotted on the x-axis, while the percentage of T cells expressing such combinations of CD107a, IFNγ, TNFα and IL2 in their response against MOMP_282–290_ (**upper** graphs) and MOMP_200–209_ (**lower** graphs) peptides are plotted on the y-axis. Responses are grouped and color-coded according to the number of functions (1, 2, 3, or 4 cytokines co-expressed). Results are presented as box and whisker plots. Each point represents one mouse, the boxes represent the interquartile range, the center line in the box represents the median, and the whiskers extend to the minimum and maximum data values (**B**) Individual multifunctional CD8^+^ T cell populations specific to MOMP_282–290_ (upper graphs) and MOMP_200–209_ (lower graphs) were plotted to compare the difference in CD8^+^ T cell magnitude between MVA-spCTH522 and MVA-CTH522:B7 immunized mice. For statistical analysis, Mann–Whitney test was used for comparing the multifunctional CD8^+^ T-cell populations (ns, non-significant, * *p* < 0.05).

To assess the multifunctional phenotype of MOMP_282–290_- and MOMP_200–209_-specific CD8^+^ T cells, we used a panel of Boolean combination gates to examine the co-expression of IFNγ, IL2, TNFα, and CD107 in splenocytes from prime-boosted A2.DR1 mice after pulsing with MOMP_282–290_ and MOMP_200–209_ peptides (Supplementary Figure S4). Both MVA-spCTH522 and MVA-CTH522:B7 induced similar multifunctional CD8^+^ T-cell populations, including MOMP_282–290_- and MOMP_200–209_-specific CD8^+^ T cells that were singly positive for IFNγ, TNFα, or CD107. We also identified CD8^+^ T-cell populations that were double-positive for IFNγ and TNFα or IFNγ and CD107, as well as a triple-positive population expressing IFNγ, TNFα, and CD107 (Figure 6A). Notably, the magnitude of these multifunctional populations was largely comparable between the two vaccine candidates, with the exception of a significantly higher percentage of IFNγ and TNFα double-positive MOMP_200–209_-specific CD8^+^ T cells in the MVA-spCTH522-immunized mice compared to the MVA-CTH522:B7-immunized mice (Figure 6B).

## 4. Discussion

*C. trachomatis* causes prevalently genital infections and can lead to serious complications such as pelvic inflammatory disease and infertility. Despite efforts to screen and treat with antibiotics, infection rates continue to rise, highlighting the need for improved solutions [1,2,3]. In light of these challenges, vaccine development is emerging as a promising strategy to control *C. trachomatis* infection [47].

MOMP is the most extensively studied protein in the field of vaccine development against *C. trachomatis*. The chlamydial MOMP has an approximate size of 40 kDa and is characterized by five genetically conserved domains and four variable domains used for serovar identification within each species. These domains contain numerous human T-cell and B-cell epitopes [48]. MVA-based vaccines are renowned for their ability to induce robust T-cell and antibody responses against recombinant antigens [46,49]. Therefore, the combination of MVA-based vaccine and MOMP-based antigen, such as CTH522, may be an optimal tool to investigate the protective role of vaccine-induced T cells and antibodies in *C. trachomatis* infection.

Our research focused on characterizing the immunogenicity of MVA-based vaccines expressing spCTH522 and CTH522:B7. Our results showed that MVA-spCTH522 and MVA-CTH522:B7 elicited different immune responses in different mouse models. Both vaccine candidates induced IFNγ-producing CD4^+^ T cells in C57BL/6J mice. Surprisingly, neither vaccine was able to induce CTH522-specific CD8^+^ T-cell responses. In C57BL/6J mice, only a few Chlamydia antigens have been identified that contain MHC-I-restricted immunodominant epitopes, such as the cysteine-rich protein antigen, CrpA [50,51]. Interestingly, a vaccinia virus-based vaccine has been shown to induce CrpA-specific effector memory CD8^+^ T cells in C57BL/6J mice [27].

Recent data on the immune response to the protein-based vaccine CTH522 in both humans and mice showed strong cytokine production, primarily IFNγ and IL-17. However, ICS analysis revealed that these cytokines are predominantly produced by CD4^+^ T cells [19]. This led to the consideration of MVA-based vaccines as a promising new strategy for infection prevention. However, the increasing importance of CD8^+^ T cells in the context of chlamydial infection is noteworthy [21]. Our results show that MVA-expressing CTH522 antigens failed to induce MOMP-specific CD8^+^ T cells in C57BL/6J mice, in contrast to A2.DR1 mice where both MVA-spCTH522 and MVA-CTH522:B7 induced robust CD8^+^ T-cell responses. While this demonstrates the ability of MVA-based vaccines to elicit T cells against recombinant antigens, our findings unveil a significant barrier regarding the MOMP antigen in C57BL/6J mice. We deduced that *C. trachomatis* MOMP may lack sufficient immunogenicity in C57BL/6J mice compared to the CrpA antigen. Such a limitation could potentially affect the ability of a MOMP-based vaccine to stimulate an efficient CD8^+^ T-cell response in this mouse model. This leads to speculation that previous preclinical studies using C57BL/6J to investigate MOMP-based vaccines may have underestimated the contribution of CD8^+^ T cells to infection clearance. Our findings highlight the potential of Chlamydia-specific CD8^+^ T cells in a mouse model that partly replicates the CD8^+^ T-cell response in humans, such as the A2.DR1 mouse model, and further support the need for in-depth investigations.

The A2.DR1 mouse model, using HLA-A*02:01/Db for MHC-I and HLA-DR1 for MHC-II [39], proved to be a valuable tool for delineating HLA-restricted epitopes in both viral and bacterial diseases, and it has been instrumental in evaluating the T cell-specific immunogenicity of vaccines [52,53,54,55,56,57,58]. Our results demonstrate that recombinant MVA-expressing engineered CTH522 antigens can induce Th1-biased CD4^+^ T cells and multifunctional CD8^+^ T cells in this mouse model, which allows us to investigate the role of MHC-dependent T cells in clearing infection and influencing the immunopathology induced by chlamydial infection. Our results not only validate the importance of the A2.DR1 mouse model for studying vaccine-induced CD8^+^ T-cell responses against HLA-restricted epitopes but also highlight its potential for uncovering novel pathogen epitopes. Both could be important for the design of antigens and vaccine development. Indeed, the identification of the MOMP_200–209_ as a novel HLA-A*02:01-restricted epitope within the chlamydial MOMP, which needs to be confirmed in humans, further underlines the importance of our approach in deciphering immune responses to *C. trachomatis*.

However, while T cells play a role in eliminating infected cells, antibodies are critical for neutralizing the elementary bodies in the mouse infection model with *C. trachomatis* [59]. Our data demonstrate that one of the two MVA-based vaccines could induce antibody responses against *C. trachomatis* antigens in both C57BL/6J and A2.DR1 mice. Although both MVA-spCTH522 and MVA-CTH522:B7 expressed high levels of antigen in vitro, only the cell-surface-anchored CTH522:B7 antigen elicited a strong antibody response. This highlights the importance of characterizing the antigens throughout the vaccine development and characterization process by evaluating antigen localization. Importantly, in C57BL/6J mice, MVA-CTH522:B7 induced a humoral response with a predominance of IgG2b and IgG2c rather than IgG1, which was associated with MOMP-based vaccine-induced protection against *C. trachomatis* infection in previous studies [60].

Our systemic vaccination approach showed that MVA-based vaccines are able to induce T-cell responses in the spleen and antibody responses in the serum. However, it is important to note that tissue-resident T cells and mucosal antibodies play a critical role in protection against Chlamydia [18,61]. Previous studies have shown that mucosal vaccination of mice with UV-inactivated *C. trachomatis* can induce protective tissue-resident CD4^+^ T cells [62]. On the other hand, parenteral vaccination with the CTH522/CAF01 formulation has also been shown to induce tissue-resident CD4^+^ T cells that are protective against *C. trachomatis* infection [63]. This suggests that different routes of vaccination may induce tissue-resident T cells. MVA-based vaccines are renowned for their ability to induce robust protective tissue-resident T-cell responses against recombinant antigens [64,65]. It remains to be determined whether other routes of vaccination with the two MVA-based vaccines in this study can induce MOMP-specific tissue-resident CD4^+^ T cells. However, the role of tissue-resident CD8^+^ T cells in Chlamydia infection is not well understood [66]. Our results suggest that the A2.DR1 mouse model may be valuable in investigating the role of both MOMP-specific CD4^+^ and CD8^+^ T cells induced by our MVA-based vaccines and their contribution to protection against *C. trachomatis* infection.

Overall, our findings underscore the complexity of vaccine development against *C. trachomatis* and highlight the importance of thoroughly studying all aspects of vaccine-induced immune responses to understand their protective role against infection. We present innovative approaches to study different components of the immune response against *C. trachomatis.* In the C57BL/6J mouse model, the MVA-spCTH522 vaccine may clarify the role of CD4^+^ T cells alone in protection against *C. trachomatis*, while the MVA-CTH522:B7 vaccine may reveal the contributions of both CD4^+^ T cells and antibody responses. Conversely, in the A2.DR1 mouse model, MVA-spCTH522 vaccination may help elucidate the role of CD4^+^ and CD8^+^ T-cell responses, while MVA-CTH522:B7 may determine whether the combined involvement of CD4^+^ T cells, CD8^+^ T cells, and antibody responses is critical for protection against infection. These approaches would be invaluable in gaining a clearer understanding of how to design an appropriate vaccine that elicits the most effective immune response to protect against *C. trachomatis* infection.

Sequence analysis of MOMP (Supplementary Table S2) across different Chlamydia species revealed that MOMP_200–209_ and MOMP_282–290_ epitopes are not only present in different serovars of *C. trachomatis* but also extend to other species such as *Chlamydia suis* and *Chlamydia muridarum*. In addition, MOMP_282–290_ is also present in *Chlamydia abortus*, *Chlamydia gallinacea*, *Chlamydia pecorum*, and *Chlamydia psittaci*. Among the different serovars of *C. trachomatis*, MOMP_200–209_ and MOMP_282–290_ epitopes are found in serovars A, B, Ba and C of *C. trachomatis*, which are the major causative agents of ocular trachoma. Trachoma is a major cause of blindness and visual impairment, with 0.4 and 1.6 million cases reported in 2015, respectively [67]. Many efforts are underway to develop a vaccine to prevent ocular trachoma [68,69,70]. In addition, these epitopes are present in the MOMP of serovars L1, L2, and L3 of *C. trachomatis*, also known as the *lymphogranuloma venereum* (LGV) biovar. Serovars L1–3 are responsible for causing invasive urogenital and anorectal infections, with the ability to invade and replicate in regional lymph nodes. Individuals with LGV may present with genital ulcers, swollen inguinal lymph nodes, or proctitis [5]. *C. abortus* primarily infects ruminants and poses a potential risk of transmission to humans. Although human infection with *C. abortus* is rare, the consequences are serious. There have been documented cases of pregnant women testing positive for *C. abortus*, resulting in pelvic inflammatory disease, placental dysfunction, and late fetal death [71,72]. The presence of these epitopes in different strains of Chlamydia highlights the zoonotic aspect of cellular-mediated cross-protective immunity elicited by recombinant MVAs expressing CTH522 antigens.

Of particular interest is that the epitopes identified are also present in the MOMP of *C. muridarum*, a Chlamydia strain infecting mice. In mice, vaginal inoculation of *C. muridarum* results in hydrosalpinx, fibrosis, and infertility, which closely resemble the common post-infection sequelae seen in women infected with *C. trachomatis* [73]. Conversely, intravaginal inoculation of *C. trachomatis* in mice typically leads to a mild genital tract infection that resolves relatively quickly. Post-infection complications only arise when high doses of *C. trachomatis* are directly inoculated into specific sites like the uterus, uterine horns, or ovarian bursa [74], thus deviating from the natural route of infection. Therefore, assessing the effectiveness of a *C. trachomatis* vaccine in protecting mice against vaginal infection with the bacterium seems challenging. This challenge may be overcome by CD8^+^ T cells that recognize short-conserved epitopes across different Chlamydia species, providing an opportunity to explore the protective capacity of the MVA-based *C. trachomatis* vaccines of this study in the context of natural vaginal infection with *C. muridarum* in A2.DR1 mice.

## 5. Conclusions

Our research contributes to the understanding of how to improve vaccine development to elicit targeted immune responses. This study highlights the importance of antigen design and selection of appropriate animal models to investigate the immune response of *C. trachomatis* vaccine candidates. Whether the immune responses observed in our mouse models will correlate with those needed for actual protection against Chlamydia in humans, and whether our vaccine candidates could be combined with the existing adjuvanted CTH522 subunit vaccine, remains to be determined through further studies. In addition, the investigation of different clinically relevant routes of immunization—such as mucosal vaccination or a combination of intramuscular and mucosal (intranasal, intravaginal and rectal) approaches—would help to clarify the ability of our MVA-based vaccine to induce both mucosal humoral responses and tissue-resident T cells. This would also allow us to assess the efficacy of these routes in protecting against *C. trachomatis*. However, it is important to note that vaginal infection with *C. trachomatis* in mice does not result in ascending infection or tubal pathology, as seen in human Chlamydia infections. The translation of data obtained in mouse models to the human immune system is limited. Therefore, testing our MVA-based vaccine in alternative animal models that mimic both the human immune response and the progression of chlamydial infection more closely, such as non-human primates, would be valuable to confirm our immunological findings and to evaluate vaccine-induced protection against *C. trachomatis* infection [75,76]. This approach holds promise for improving vaccine efficacy to protect against *C. trachomatis* and across different Chlamydia species, bringing us closer to formulating effective immunization strategies against this persistent and widespread pathogen.

## Data Availability

The authors confirm that all relevant data are included in the paper and are available on request from the corresponding author.

## Supplementary Materials

The following supporting information can be downloaded at: https://www.mdpi.com/article/10.3390/vaccines12080944/s1, Table S1: Amino acid sequences of CTH522, spCTH522 and CTH522:B7 antigens; Figure S1: Construction and genetic and functional characterization of MVA-CTH522; Figure S2: CTH522:B7 is located at the cell surface membrane; Figure S3: Single dose and prime/boost vaccination regimens with MVA-spCTH522 and MVA-CTH522:B7 induce MVA-specific CD8^+^ and CD4^+^ T cell responses; Figure S4: Gating strategy for the analysis of epitope-specific multifunctional CD8^+^ T cells in the spleen; Table S2: Presence of HLA-A*02:01 restricted MOMP-derived peptide epitopes in *Chlamydia* species.
