# Supplementary material for: Viral Vector-Based Chlamydia trachomatis Vaccines Encoding CTH522 Induce Distinct Immune Responses in C57BL/6J and HLA Transgenic Mice
Source: Vaccines (Basel). 2024 Aug 22;12(8):944. doi: 10.3390/vaccines12080944 (PMC11360449; doi:10.3390/vaccines12080944)
Supplement: Supplementary file 1 [file vaccines-12-00944-s001.zip › vaccines-3126808-supplementary.pdf]

Supplementary data

>Aminoacid sequence of CTH522

MGDAISMRVGYYGDFVFDRVLKTDVNKEFQMGAKPTTDTGNSAAPSTLT
ARENPAYGRHMQDAEMFTNAACMALNIWDRFDVFCTLGATSGYLKGNSA
SFNLVGLFGDNENQKTVKAESVPNMSFDQSVVELYTDTTFAWSVGARAAL
WECGCATLGASFQYAQSKPKVEELNVLCNAAEFTINKPKGYVGKEFPLDL
TAGTDAATGTKDASIDYHEWQASLALSYRLNMFTPYIGVKWSRASFDADT
IRIAQPKSATAIFDTTTLNPTIAGAGDVKTGAEGQLGDTMQIVSLQLNNMFT
PYIGVKWSRASFDADTIRIAQPKSATAIFDTTTLNPTIAGAGDVKASAEGQL
GDTMQIVSLQLNNMFTPYIGVKWSRASFDSDTIRIAQPRLVTPVVDITTLNP
TIAGCGSVAGANTEGQISDTMQIVSLQLNNMFTPYIGVKWSRASFDSNTIRI
AQPKLAKPVVDITTLNPTIAGCGSVVAANSEGQISDTMQIVSLQLN*

>Aminoacid sequence of spCTH522

MKKLLKSVLVFAALSSASSLQALPVGNPAEPSLMIDGILWEGFGGDPCDPC
ATWCDAISMRVGYYGDFVFDRVLKTDVNKEFQMGAKPTTDTGNSAAPST
LTARENPAYGRHMQDAEMFTNAACMALNIWDRFDVFCTLGATSGYLKGN
SASFNLVGLFGDNENQKTVKAESVPNMSFDQSVVELYTDTTFAWSVGARA
ALWECGCATLGASFQYAQSKPKVEELNVLCNAAEFTINKPKGYVGKEFPL
DLTAGTDAATGTKDASIDYHEWQASLALSYRLNMFTPYIGVKWSRASFDA
DTIRIAQPKSATAIFDTTTLNPTIAGAGDVKTGAEGQLGDTMQIVSLQLNNM
FTPYIGVKWSRASFDADTIRIAQPKSATAIFDTTTLNPTIAGAGDVKASAEG
QLGDTMQIVSLQLNNMFTPYIGVKWSRASFDSDTIRIAQPRLVTPVVDITTL
NPTIAGCGSVAGANTEGQISDTMQIVSLQLNNMFTPYIGVKWSRASFDSNTI
RIAQPKLAKPVVDITTLNPTIAGCGSVVAANSEGQISDTMQIVSLQLN*

>Amino acid sequence of CTH522:B7

METDTLLLWVLLLWVPGSTGDGDAISMRVGYYGDFVFDRVLKTDVNKEF
QMGAKPTTDTGNSAAPSTLTARENPAYGRHMQDAEMFTNAACMALNIWD
RFDVFCTLGATSGYLKGNSASFNLVGLFGDNENQKTVKAESVPNMSFDQS
VVELYTDTTFAWSVGARAALWECGCATLGASFQYAQSKPKVEELNVLCN
AAEFTINKPKGYVGKEFPLDLTAGTDAATGTKDASIDYHEWQASLALSYRL
NMFTPYIGVKWSRASFDADTIRIAQPKSATAIFDTTTLNPTIAGAGDVKTGA
EGQLGDTMQIVSLQLNNMFTPYIGVKWSRASFDADTIRIAQPKSATAIFDTT

TLNPTIAGAGDVKASAEGQLGDTMQIVSLQLNNMFTPYIGVKWSRASFDS DTIRIAQPRLVTPVVDITTLNPTIAGCGSVAGANTEGQISDTMQIVSLQLNN MFTPYIGVKWSRASFDSNTIRIAQPKLAKPVVDITTLNPTIAGCGSVVAANS EGQISDTMQIVSLQLNPPEDPPDSKNTLVLFGAGFGAVITVVVIVVIIKCFCK HRSCFRRNEASRETNNSLTFGPEEALAEQTVFL*

Supplementary Table 1. Amino acid sequences of CTH522, spCTH522 and CTH522:B7 antigens

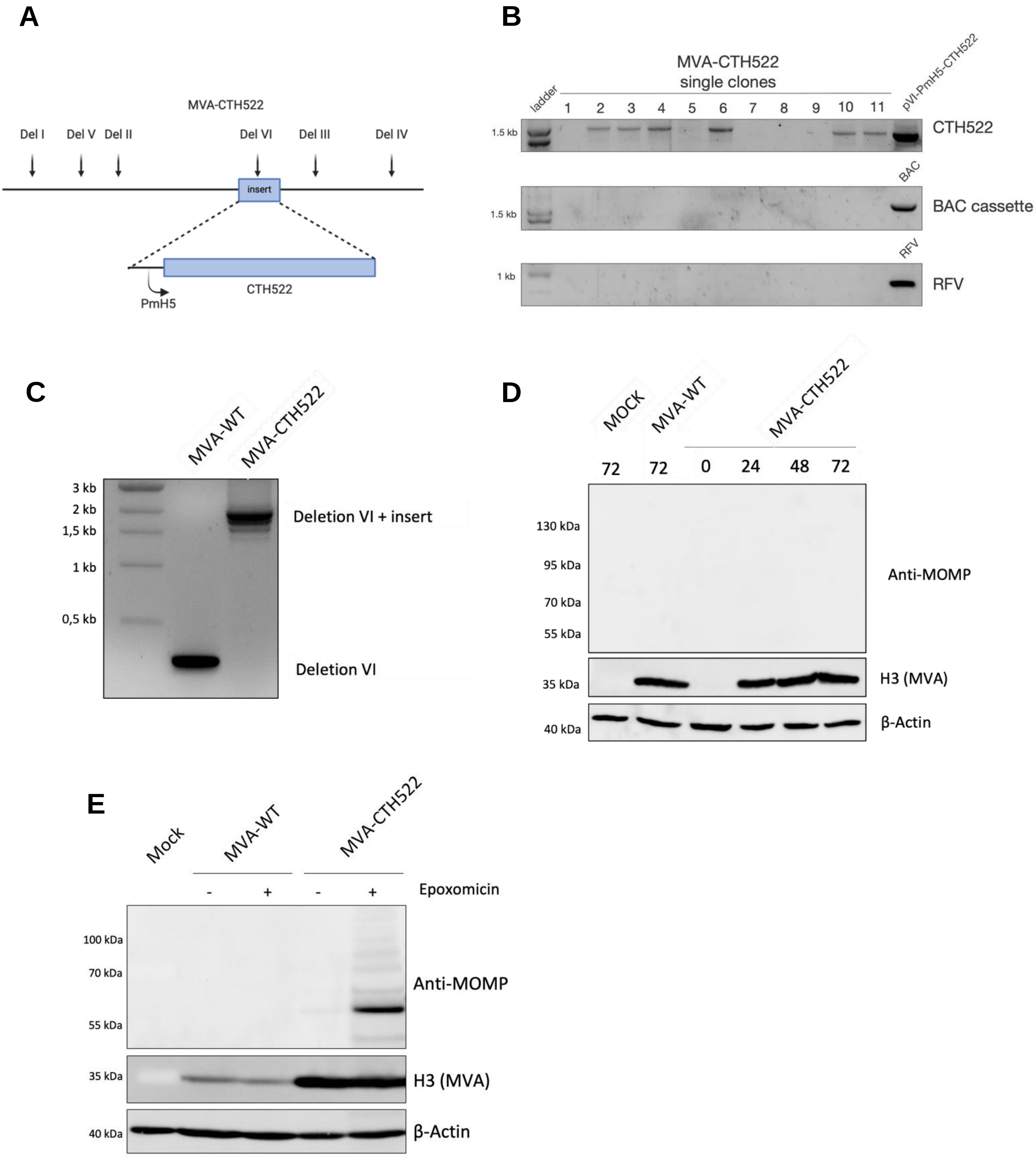

Supplementary Figure 1. Construction and genetic and functional characterization of MVA-CTH522.

(A) Schematic representation of recombinant MVA-CTH522 genome. The location of the transgene (insert) in the major deletion site VI of the MVA genome is shown at the top. PmH5, modified early late vaccinia virus promoter H5. (B) DNA was extracted

from MVA-CTH522 infected DF-1 cells seeded in a 96-well plate in limiting dilutions. Single clones were screened and selected (1 to 11) when single plaques were present (cytopathic effect). PCR was carried out using primers specific for either delVI containing CTH522, the RFV genome or GFP containing BAC cassette. MVA transfer plasmid pVI-PmH5-CTH522, viral genomic DNA extracted from RFV, and the purified recombinant MVA-BAC were used as control. (C) Viral genomic DNA was extracted from purified stocks of MVA-WT or MVA-CTH522 and analyzed by PCR using delVI-specific primers. The size of the expression cassette was expected to be 1986 bp for CTH522 and 306 bp for MVA-WT. (D) HeLa cells were mock-infected or infected with MVA-WT or MVA-CTH522 (MOI = 5). Cells were lysed at 0, 24, 48 or 72 hpi (MVA-CTH522) or at 72 hpi (mock and MVA-WT). Whole cell lysates were analyzed by Western blotting using antibodies against MOMP-SvD expecting a protein fraction of ~53,5 kDa for CTH522. Vaccinia virus H3 protein and b-actin were used as infection and loading controls, respectively. (E) HeLa cells were mock-infected or infected with MVA-WT or MVA-CTH522 (MOI = 5). At 3 hpi, cells were treated with 2 uM of Epoxomicin or DMSO. Whole cell lysates were collected at 24 hpi. Protein extracts were analyzed by Western blotting with antibodies against MOMP-SvD demonstrating a protein with an expected size of ~53,5 kDa for CTH522. Vaccinia virus H3 protein and b-actin were used as infection and loading controls, respectively.

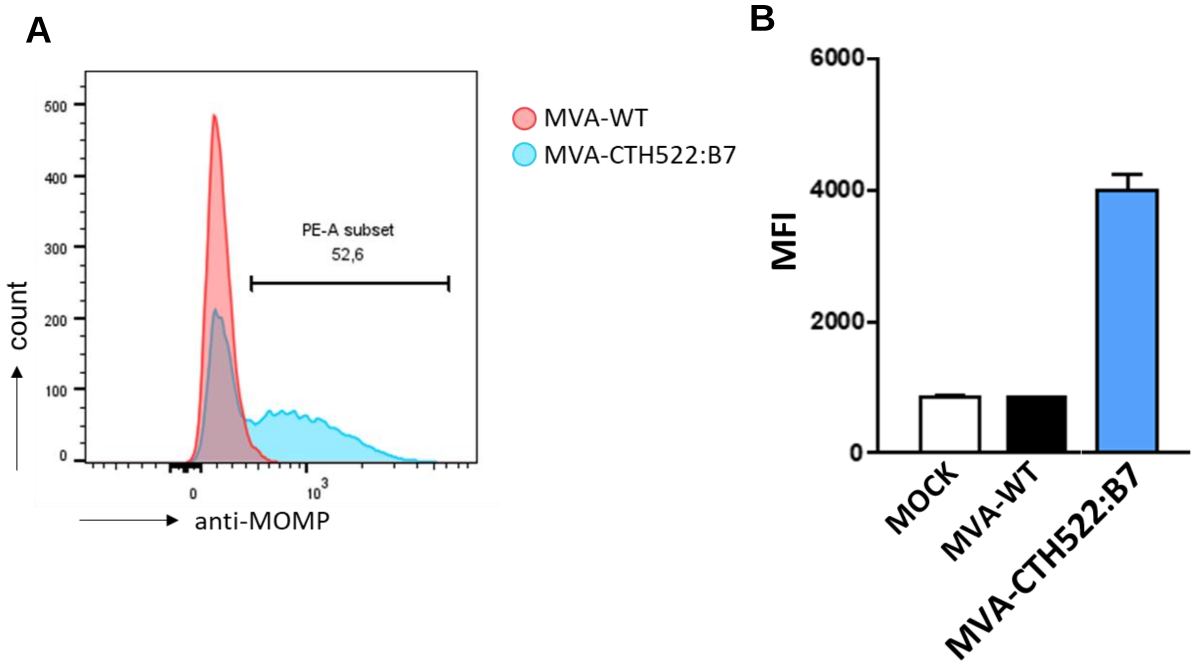

Supplementary Figure 2. CTH522:B7 is located at the cell surface membrane. Flow cytometry analysis to determine the location of CTH522:B7 proteins in HeLa cells infected with MVA-CTH522:B7 or MVA-WT at MOI of 5. Non-permeabilized were stained with a mouse monoclonal anti-MOMP antibody. Bound anti-MOMP was visualized using a mouse secondary antibody conjugated to phycoerythrin (PE). (A) Representative histogram gating to determine the mean fluorescence intensity (MFI) of the surface exposed CTH522:B7 antigen (y-axis). Pink, MVA-WT infected cells; pale blue, MVA-CTH522:B7 infected cells. (B) Mean of MFI (+/-SD) of PE (CTH522) in Mock, MVA-WT, or MVA-CTH522:B7 infected cells.

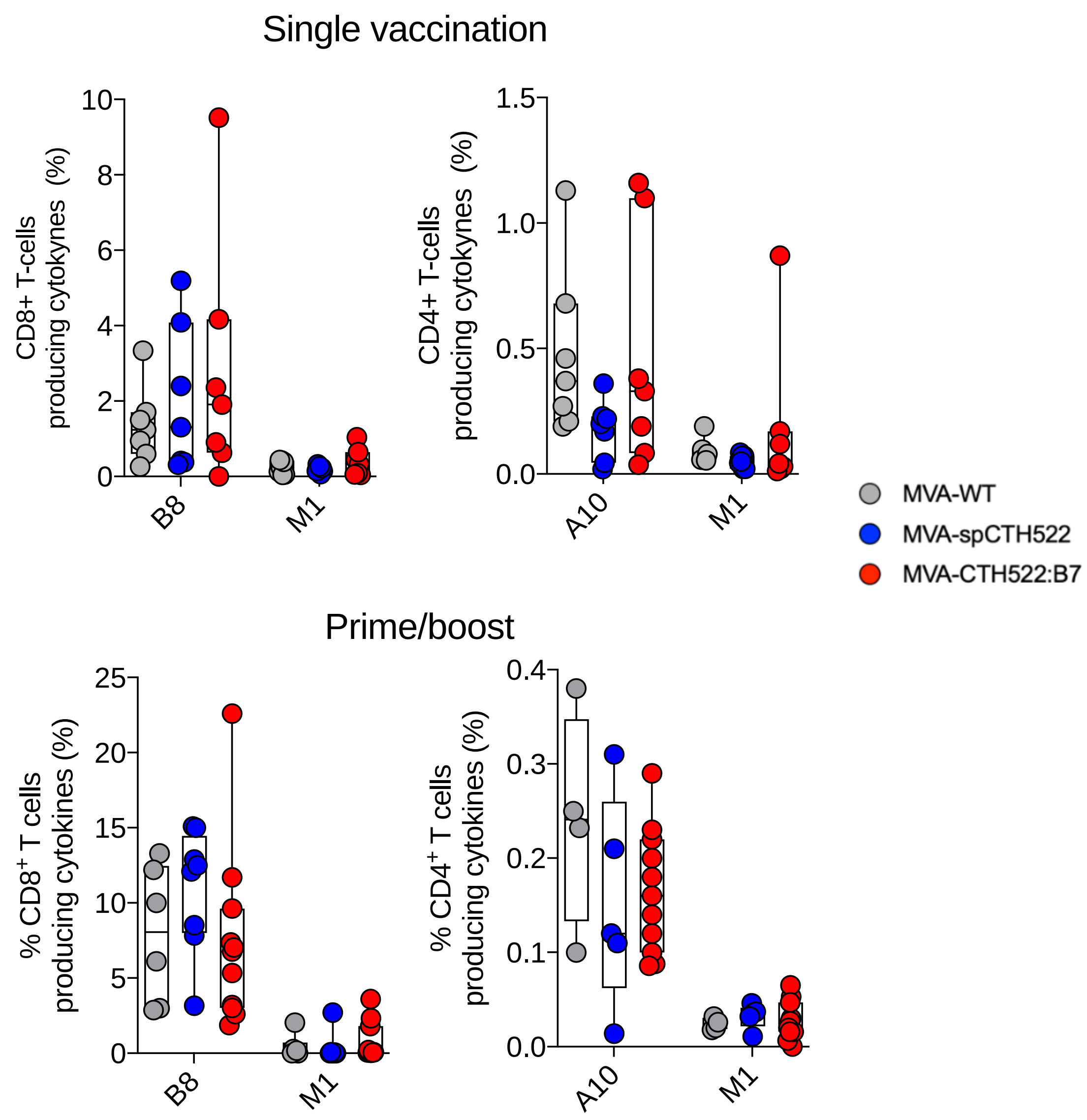

Supplementary Figure 3. Single dose and prime/boost vaccination regimens with MVA-spCTH522 and MVA-CTH522:B7 induce MVA-specific CD8$^+$ and CD4$^+$ T cell responses.

A2.DR1 mice were immunized intraperitoneally with either a single vaccination (as in Fig. 4A) or a prime/boost regimen (as in Fig. 5A) using MVA-WT, MVA-spCTH522, or MVA-CTH522:B7. Splenocytes from immunized mice were restimulated with MVA derived B8$_{20–27}$ or A10$_{293-307}$ peptides for CD8$^+$ or CD4$^+$ T cell activation, respectively. Cytokine production (IFNγ, TNFα, CD107, IL2) was measured by ICS assay followed by FACS analysis. Gating strategy is displayed in Supplementary Figure 3. Peptides derived from Matrix protein 1 (M1) of Influenza virus were used as negative controls for CD8$^+$ (M1$_{58-66}$) and CD4$^+$ T (M1$_{17-30}$) cell responses.

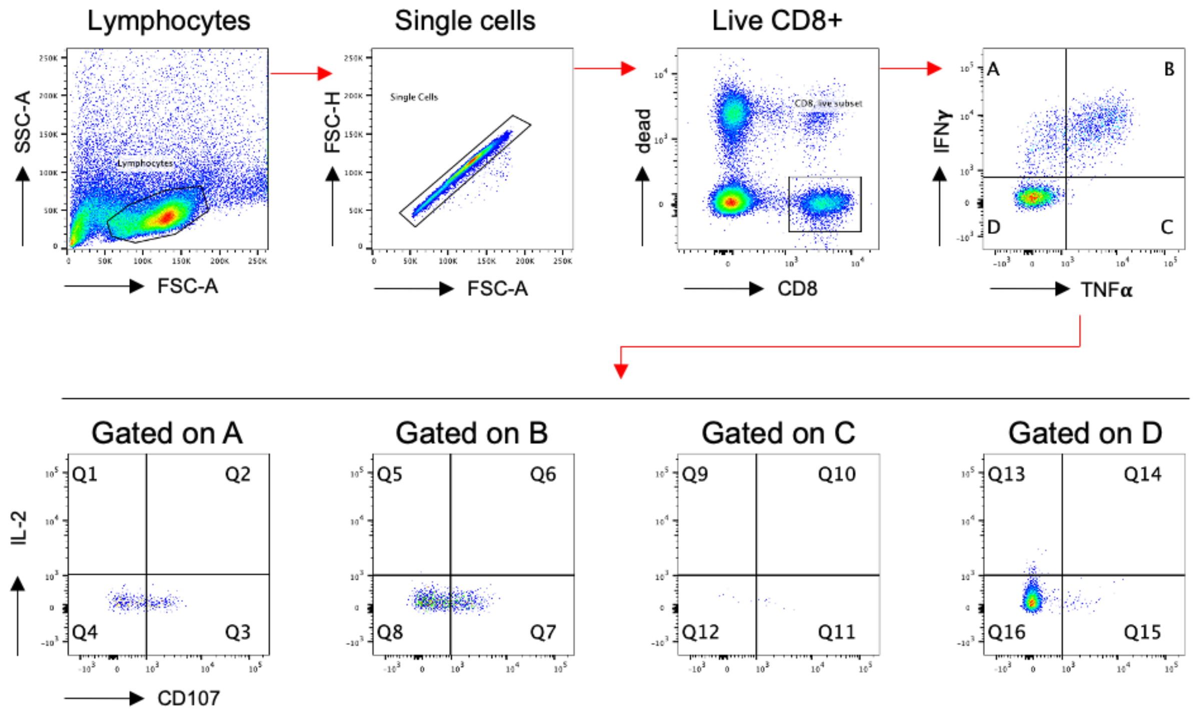

- Q1: IFNγ $^{+}$,TNFα^{-}, CD107^{-}, IL2^{+}
- Q2: IFNγ $^{+}$,TNFα^{-}, CD107^{+}, IL2^{+}
- Q3: IFNγ $^{+}$,TNFα^{-}, CD107^{+}, IL2^{-}
- Q4: IFNγ $^{+}$,TNFα^{-}, CD107^{-}, IL2^{-}
- Q5: IFNγ $^{+}$,TNFα^{+}, CD107^{-}, IL2^{+}
- Q6: IFNγ $^{+}$,TNFα^{+}, CD107^{+}, IL2^{+}
- Q7: IFNγ $^{+}$,TNFα^{+}, CD107^{+}, IL2^{-}
- Q8: IFN^{+},TNFα^{+}, CD107^{-}, IL2^{-}
- Q9: IFNγ $^{-}$,TNFα^{+}, CD107^{-}, IL2^{+}
- Q10: IFNγ $^{-}$,TNFα^{+}, CD107^{+}, IL2^{+}
- Q11: IFNγ $^{-}$,TNFα^{+}, CD107^{+}, IL2^{-}
- Q12: IFNγ $^{-}$,TNFα^{+}, CD107^{-}, IL2^{-}
- Q13: IFNγ $^{-}$,TNFα^{-}, CD107^{-}, IL2^{+}
- Q14: IFNγ $^{-}$,TNFα^{-}, CD107^{+}, IL2^{+}
- Q15: IFNγ $^{-}$,TNFα^{-}, CD107^{+}, IL2^{-}
- Q16: IFNγ $^{-}$,TNFα^{-}, CD107^{-}, IL2^{-}

Supplementary Figure 4. Gating strategy for the analysis of epitope-specific multifunctional CD8^{+} T cells in the spleen. Splenocytes from MVA-CTH522:B7 immunized mice following the prime/boost regimen (as in Fig. 5A) were restimulated in vitro with MOMP$_{282-290}$ for 5-6h. (Upper plots) After ICS, splenocytes were analyzed by flow cytometry gating on lymphocytes, single cells, and CD8 live cells. CD8^{+} T cells were then plotted on IFNγ (x-axis) and TNFα (y-axis) production, resulting in four populations, A (IFNγ^{+}, TNFα^{-}), B (IFNγ^{+}, TNFα^{+}), C (IFNγ^{-}, TNFα^{+}), and D (IFNγ^{-}, TNFα^{-}). (Lower plots) A, B, C, and D, populations were further plotted on IL2 (x-axis) and CD107 (y-axis) production. This strategy allowed to analyze all the

possible combinations of IFNγ, TNFα, IL2, and CD107, resulting in 16 different populations, Q1 to Q16. Each population was then calculated as a percentage of total CD8$^+$ live cells using FlowJo software (as in Fig. 5E).

Table 2. *Chlamydia* spp. with 100% identity of NMFTPYIGV and ALWECGCATL epitopes within MOMP*

NMFTPYIGV	ALWECGCATL
Chlamydia abortus	Chlamydia muridarum
Chlamydia gallinacea	Chlamydia suis
Chlamydia muridarum	Chlamydia trachomatis
Chlamydia pecorum	Chlamydia trachomatis 434/Bu
Chlamydia psittaci	Chlamydia trachomatis A/363
Chlamydia suis	Chlamydia trachomatis A/5291
Chlamydia trachomatis	Chlamydia trachomatis A/7249
Chlamydia trachomatis C/TW-3	Chlamydia trachomatis A/HAR-13
Chlamydia trachomatis L3/404/LN	Chlamydia trachomatis A2497
Chlamydia trachomatis A2497	Chlamydia trachomatis B/Jali20/OT
Chlamydia trachomatis A/363	Chlamydia trachomatis B/TZ1A828/OT
Chlamydia trachomatis A/5291	Chlamydia trachomatis C/TW-3
Chlamydia trachomatis A/7249	Chlamydia trachomatis D/UW-3/CX
Chlamydia trachomatis A/HAR-13	Chlamydia trachomatis E/CS88
Chlamydia trachomatis G/9768	Chlamydia trachomatis F/SotonF3
Chlamydia trachomatis G/11074	Chlamydia trachomatis G/11222
Chlamydia trachomatis G/9301	Chlamydia trachomatis G/9301
Chlamydia trachomatis G/11222	Chlamydia trachomatis G/9768
Chlamydia trachomatis G/SotonG1	Chlamydia trachomatis G/SotonG1
Chlamydia trachomatis B/Jali20/OT	Chlamydia trachomatis L1/115
Chlamydia trachomatis L2/434/Bu	Chlamydia trachomatis L1/1322/p2
Chlamydia trachomatis L2c	Chlamydia trachomatis L1/224
Chlamydia trachomatis B/TZ1A828/OT	Chlamydia trachomatis L1/440/LN
Chlamydia trachomatis L1/224	Chlamydia trachomatis L2c
Chlamydia trachomatis D/UW-3/CX	Chlamydia trachomatis L3/404/LN
Chlamydia trachomatis L1/115	
Chlamydia trachomatis L1/440/LN	
Chlamydia trachomatis L1/1322/p2	

*based on the BLAST tool in NCBI Entrez Protein Database (nr)

Supplementary Table 2. Presence of HLA-A*02:01 restricted MOMP-derived peptide epitopes in *Chlamydia* species
